# Protocol to assess arteriovenous differences across the liver and hindlimb muscles in mice following treadmill exercise

**DOI:** 10.1016/j.xpro.2022.101985

**Published:** 2023-01-03

**Authors:** Stephen P. Ashcroft, Sara L. Jepsen, Amy M. Ehrlich, Jonas T. Treebak, Jens J. Holst, Juleen R. Zierath

**Affiliations:** 1 Novo Nordisk Foundation Center for Basic Metabolic Research, Faculty of Health and Medical Sciences, University of Copenhagen, Copenhagen, Denmark; 2 Department of Biomedical Sciences, Faculty of Health and Medical Sciences, University of Copenhagen, Copenhagen, Denmark; 3 Deparment of Molecular Medicine and Surgery, Integrative Physiology, Karolinska Institutet, Stockholm, Sweden; 4 Department of Physiology and Pharmacology, Integrative Physiology, Karolinska Institutet, Stockholm, Sweden

**Keywords:** Behavior, Health Sciences, Metabolism, Model Organisms

## Abstract

The tissue-specific release and uptake of metabolites in response to exercise is incompletely understood. Here, we detail a protocol to assess arteriovenous differences across the liver and hindlimb muscles in response to treadmill exercise in mice. We describe steps for the treadmill running of mice and the region-specific sampling of blood from the liver and hindlimb. This procedure is particularly relevant for the study of tissue-specific metabolism in response to exercise.

For complete details on the use and execution of this protocol, please refer to Sato et al. (2022).^1^

## Highlights

• Protocol for arteriovenous blood sampling from mouse liver and hindlimb after exercise
• Surgical techniques, cannulations, and blood sampling in mouse models
• Conditions for treadmill running, including slope, speed, and duration
• Samples can be utilized to quantify the flux of metabolites from liver and muscle

## Abstract (editor highlights)

Publisher’s note: Undertaking any experimental protocol requires adherence to local institutional guidelines for laboratory safety and ethics.

## Graphical abstract

**Figure:**
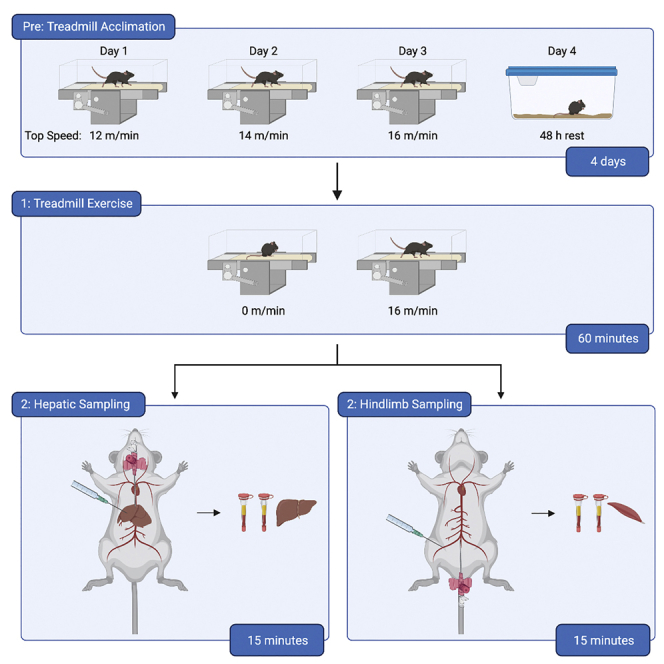

## Before you begin

The protocol described below contains the specific steps to perform arteriovenous sampling of blood from the liver and hindlimb following a single bout of treadmill exercise in mice. We have successfully utilized this protocol to investigate the time- and tissue-specific responses to treadmill running in mice.^1^ Regardless of whether one intends to study the time-of-day-specific responses to exercise in particular, it is important to consider at which time the exercise bout is performed. We and others have previously reported time-of-day-dependent changes in maximal exercise performance, as well as the transcriptomic and metabolomic responses to acute exercise.^2^^,^^3^ Furthermore, detailed information regarding daytime differences in moderate intensity exercise capacity has been reported.^4^

### Institutional permissions

All experiments complied with the European directive 2010/63/EU of the European Parliament and were approved by the Danish Animal Experiments Inspectorate (2012-15-2934-26 and 2015-15-0201-796). Male C57BL/6JBomTac mice were purchased from Taconic Biosciences. Before commencing with the protocol described below, it is important to ensure that ethical approval is obtained from the relevant body.

This protocol for the quantification of the net uptake and release of metabolites from either the liver or hindlimb muscle may be adapted to study responses to a variety of stimuli other than exercise. If blood sampling will not be performed post-exercise, proceed to “step-by-step method details”.

### Acclimation to treadmill

**Timing: 4 days**

Prior to the experimental day, all mice should be acclimated to the treadmill. Prior familiarization to the treadmill will reduce the stress associated with a novel stimulus for the mice.

1. Determine the slope at which to set up the treadmill.

**CRITICAL:** We have previously utilized a slope of 5°.^1^^,^^2^^,^^5^ Differences in the slope of the treadmill will affect the workload and the recruitment of muscles involved in the running exercise. Therefore, it is extremely important to pay attention to the slope before each experiment.

2. Determine the speed.

***Note:*** We have previously performed acute exercise bouts at 16–18 m/min for 1 h.^1^^,^^2^^,^^5^

**CRITICAL:** If performing a time-of-day-dependent experiment that involves working during the animal’s dark phase, it may be necessary to perform the treadmill exercise bouts under conditions of red light.

3. Day 1 of acclimation.
  a. Place mice on a stationary treadmill.
    **CRITICAL:** All mice should be acclimated to the treadmill regardless of whether they will act as sedentary or exercised mice during the experiment. We recommend that the acclimation period occurs over 3 days, with the speed of the treadmill increasing incrementally over each day.
  b. Turn on the electric shock intensity to 0.1 mA.
    ***Note:*** Most rodent treadmills are equipped with shock grids that encourage the mice to run. We perform acute exercise bouts with the settings on a mild shock (0.1 mA). For further encouragement it may be preferable to stimulate the mice via light brushing with either the hand or small bristled instrument.
  c. Allow mice to become accustomed to stationary treadmill for 5 min.
    ***Note:*** The 5 min of stationary treadmill exercise is only necessary on the first day of acclimation.
  d. Start the treadmill at 6 m/min. Maintain this speed for 2 min.
  e. Accelerate speed to 8 m/min and maintain this speed for 2 min.
  f. Accelerate speed to 10 m/min and maintain this speed for 2 min.
  g. Accelerate speed to 12 m/min and maintain this speed for a further 4 min.
  h. Stop the treadmill.
  i. Return mice to home cage.
    ***Note:*** This results in a total treadmill time of 15 min.

4. Day 2 of acclimation.
  a. Place mice on a stationary treadmill.
  b. Turn on the electric shock intensity to 0.1 mA.
  c. Start treadmill at 6 m/min and maintain this speed for 5 min.
  d. Accelerate speed to 8 m/min and maintain this speed for 2 min.
  e. Accelerate speed to 10 m/min and maintain this speed for 2 min.
  f. Accelerate speed to 12 m/min and maintain this speed for 2 min.
  g. Accelerate speed to 14 m/min and maintain this speed for a further 4 min.
  h. Stop the treadmill.
  i. Return mice to home cage.
5. Day 3 of acclimation.
  a. Place mice on a stationary treadmill.
  b. Turn on the electric shock intensity to 0.1 mA.
  c. Start treadmill at 6 m/min and maintain this speed for 5 min.
  d. Accelerate speed to 8 m/min and maintain this speed for 2 min.
  e. Accelerate speed to 10 m/min and maintain this speed for 2 min.
  f. Accelerate speed to 12 m/min and maintain this speed for 2 min.
  g. Accelerate speed to 14 m/min and maintain this speed for 2 min.
  h. Accelerate speed to 16 m/min and maintain this speed for a further 2 min.
  i. Stop the treadmill.
  j. Return mice to home cage.
6. Mice should be given at least 48 h rest between the final acclimation bout and the onset of the experimental bout of treadmill running.

***Note:*** It is recommended that both the acclimation and the final exercise running bout are performed at the same time of day. This will ensure that the animals are acclimated to running at a specific time and potentially reduce variation in the response to running.

### Treadmill exercise

**Timing: 60 min**

***Note:*** We have previously run one mouse on the treadmill while one mouse is on the stationary treadmill. The onset of exposure to the treadmill is then staggered by 15 min to ensure sufficient time for the blood sampling to take place and each mouse is on the treadmill for a total time of 60 min.

7. Ensure the treadmill is set up correctly according to experimental requirements.
8. Place a mouse on the exercising treadmill.
9. Turn on electric shock (0.1 mA).
10. Turn on the treadmill to 6 m/min.
11. Increase the speed by 2 m/min every 2 min until a speed of 16 m/min or another predetermined top speed is reached.
12. Once the exercising mouse has been on the treadmill for 15 min, place a mouse on the stationary treadmill.

**CRITICAL:** Mice that are acting as sedentary controls should be placed on a stationary treadmill during the same time as mice that are running. This should mitigate the influence of the stress that is associated with treadmill running in mice.

13. Allow the mouse to continue running until a total running time of 60 min is reached.

***Note:*** Throughout the exercise bout, continuously monitor the running performance of the mice. Encouragement via brushing or gently tapping the mice may be required throughout the exercise bout.

***Note:*** In some cases, mice will not cope well with treadmill running. Mice should be removed from the treadmill if they continuously spend more than 5 s on the shock grid even when further encouragement is given. If this happens during the acclimation period, mice should be excluded from the study.

14. Remove the exercising mouse from the treadmill and proceed to blood sampling.
15. Remove the mouse from the stationary treadmill 15 min later and proceed with blood sampling.

## Key resources table

**Table:** 

REAGENT or RESOURCE	SOURCE	IDENTIFIER
**Chemicals, peptides, and recombinant proteins**		

Pentobarbital (50 mg/mL)	Skanderborg Apotek	Custom
Phosphate buffered saline pH 7.2 (1×)	Gibco	Cat# 20012-019

**Deposited data**		

A/V metabolome, skeletal muscle, sedentary and exercise, ZT3 and ZT15	Sato et al.^1^	https://doi.org/10.17632/6x5vd4d5rd.1
A/V metabolome, hindlimb arterial blood, sedentary and exercise, ZT3 and ZT15	Sato et al.^1^	https://doi.org/10.17632/6x5vd4d5rd.1
A/V metabolome, hindlimb venous blood, sedentary and exercise, ZT3 and ZT15	Sato et al.^1^	https://doi.org/10.17632/6x5vd4d5rd.1
A/V metabolome, liver, sedentary and exercise, ZT3 and ZT15	Sato et al.^1^	https://doi.org/10.17632/6x5vd4d5rd.1
A/V metabolome, portal vein, sedentary and exercise, ZT3 and ZT15	Sato et al.^1^	https://doi.org/10.17632/6x5vd4d5rd.1
A/V metabolome, vena cava, sedentary and exercise, ZT3 and ZT15	Sato et al.^1^	https://doi.org/10.17632/6x5vd4d5rd.1

**Experimental models: Organisms/strains**		

10 week old male C57BL6/JBomTac mice	Taconic Biosciences	B6JBOM-M

**Other**		

Animal treadmill	Columbus Instruments	Exer 3/6
Mock animal treadmill	Columbus Instruments	Custom
Standard rodent laboratory diet	Altromin	Cat# 1310
Microvette® CB 300 K2 EDTA	Sarstedt	Cat# 16.444.100
BD Insyte™ Autoguard™ Catheter 24G × 0.75	BD	Cat# 381512
0.2 mL tube	LABSOLUTE	Cat# 7696511
1 mL syringe	NORM-JECT	Cat# 4010-200V0
25G needle	BD	Cat# 300400
27G needle	BD	Cat# 300635

## Step-by-step method details

### Hepatic blood sampling

**Timing: 15 min**

This step involves the blood sampling from the portal vein and then from a catheter inserted into the caudal vena cava at the level of the liver. Following sampling, the liver is then excised, and blood samples centrifuged to obtain plasma. Plasma samples can then be utilized to assess the net uptake and release of measured analytes to and from the liver.

***Note:*** Prior to starting the experimental protocol, a station for dissections and blood sampling should be set up with the necessary surgical and laboratory tools and reagents. In addition, a centrifuge should be set to cool to 4°C for the later spinning of blood samples obtained.

1. Load a 1 mL syringe with pentobarbital saline solution (50 mg/mL).
2. Scruff and inject the mouse intraperitoneally with pentobarbital solution (100 mg/kg).

**CRITICAL:** The correct use of anesthesia is particularly important to keep the heart continuously pumping during the blood sampling. Cervical dislocation for example, will prevent this. We have routinely used pentobarbital for this protocol, however, other anesthetics may be used. Note, however, that metabolic effects of anesthetics vary markedly.^6^

3. Return the mouse to an empty clean cage. Anesthesia should take approximately 3–5 min.
4. Once under anesthesia, place the mouse supine on the dissection station.
5. Using forceps, pinch the paw to check for a reflex response. If there is no reflex response, proceed to next step. If a response is obtained, wait until there is no response.

***Note:*** Once the animal is anesthetized, you may work under normal light conditions if previously working under red light.

**Figure 1:**
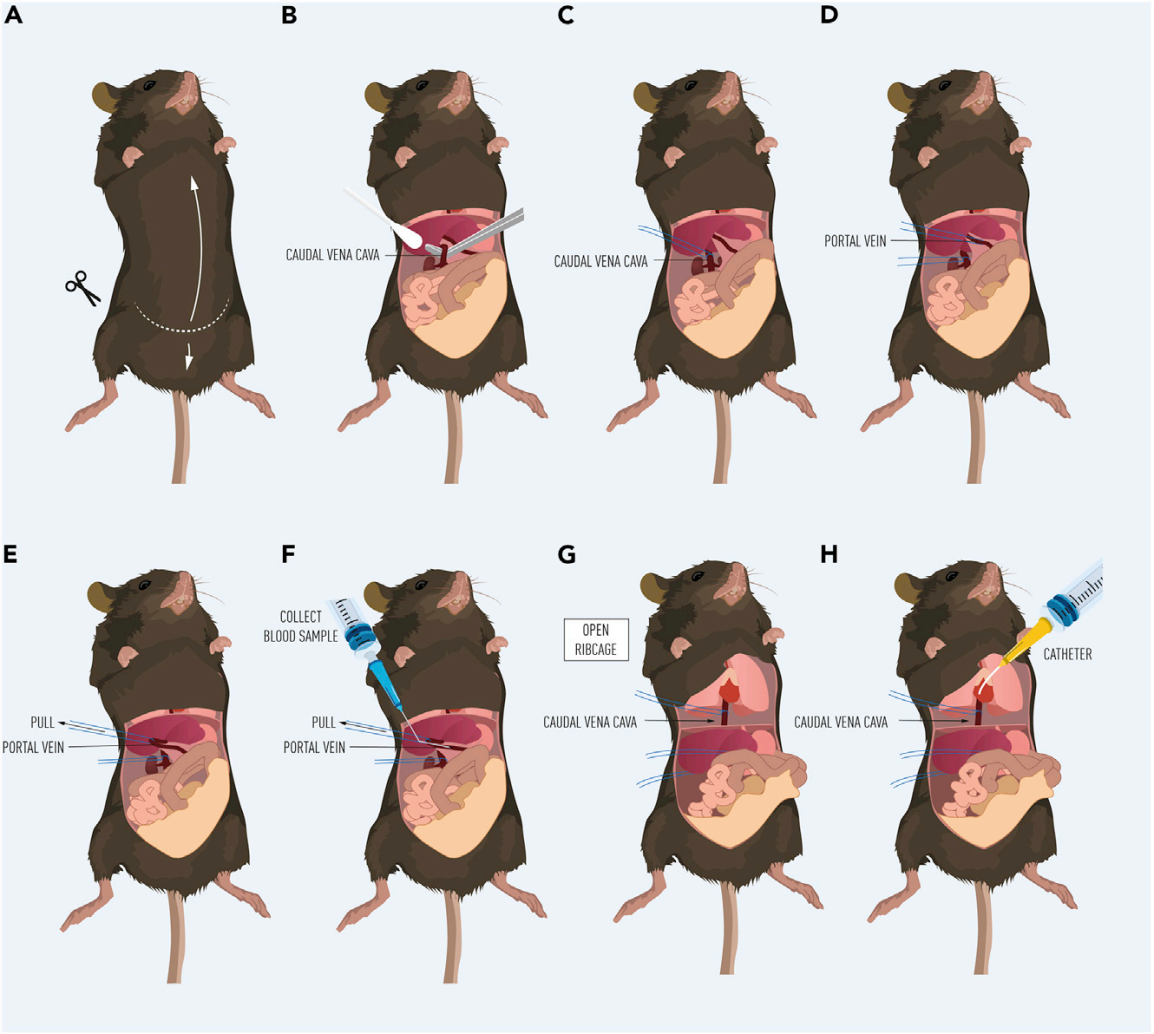
Hepatic blood sampling from the portal vein (A–F) and caudal vena cava (G–H)
7. Using a clean cotton swab, locate the caudal vena cava (Figure 1B).
8. Using curved forceps, underhook the caudal vena cava (Figure 1B) and tie a loose ligature around it above the renal veins (Figure 1C).

***Note:*** The caudal vena cava is occluded *after* the blood sampling from the portal vein to prevent blood flow from the lower extremities when taking the blood sample from the proximal part of caudal vena cava draining the liver.

6. To open the mouse, make an incision with scissors at the base of the abdomen. Cut upwards to the ribcage (Figure 1A).

9. Locate the portal vein and place a ligature around the portal vein without tying. (Figure 1D).
10. The ligature is then pulled to the right side of the mouse to further isolate the portal vein and create tension, making it easier to draw a blood sample (Figure 1E).
11. Using a 1 mL syringe and 27-gauge needle, collect a 150–160 μL blood sample from the portal vein (Figure 1F).
12. Remove the needle from the syringe and decant the blood sample into a Microvette® blood collection tube containing EDTA. Place the tube on ice.

**CRITICAL:** If there is bleeding upon removing the needle from the portal vein, it is recommended that gentle pressure is applied to the portal vein with sterile gauze in order to stop the bleeding before moving onto the next blood sample.

***Note:*** Prior to decanting, the needle is removed from the syringe to prevent hemolysis of the blood sample.

***Note:*** The Microvette® collection tube is narrow at the bottom and particularly suited to the collection of small blood samples.

13. Now tie the ligature around the inferior part of the caval vein.
14. Using scissors, cut the ribcage to expose the heart and upper part of the caudal vena cava (Figure 1G).
15. Expose the upper caudal vena cava and loosely tie a ligature around it (Figure 1G).
16. Begin inserting the catheter into the proximal caudal vena cava from the right heart chamber.
17. Following the perforation of the heart chamber by the needle, remove the needle from the catheter.
18. Further guide the catheter with the forceps down through the vena cava so the tip of the catherter is as close to the liver as possible.
19. Tie the ligature around the catheter once it is in place (Figure 1H).
20. Blood is sampled from the catheter by attaching a 1 mL syringe to draw 80–100 μL of blood from the catheter (Figure 1H).
21. Remove the syringe from the catheter and decant the blood sample into a Microvette® blood collection tube containing EDTA. Place the tube on ice.
22. Dissect the liver, rinse in phosphate buffered saline to remove excess blood and flash freeze the liver in liquid N_2_.

***Note:*** The liver may be flash frozen via the use of a freeze clamp. The use of the freeze clamp should be considered based upon what analyses will be performed downstream.

23. Spin the obtained blood samples at 2,000 g at 4°C for 10 min.
24. Remove the plasma supernatant and decant into a 0.2 mL tube. Store at −80°C for further analysis.

### Hindlimb blood sampling

**Timing: 15 min**

**CRITICAL:** This is an arterio-venous blood sampling method related to that described above for the liver. If both techniques are to be performed, then these should take place in separate cohorts of mice due to the limited amount of blood that can be collected from a mouse.

This step involves the sampling across the hindlimb via the sampling of venous blood from the right common iliac vein and arterial blood from the abdominal aorta. Following sampling, the *gastrocnemius* muscle is excised, and blood samples are subjected to centrifugation in order to obtain plasma. Plasma samples can then be used to assess the net uptake and release of measured analytes to and from the hindlimb muscles.

**Figure 2:**
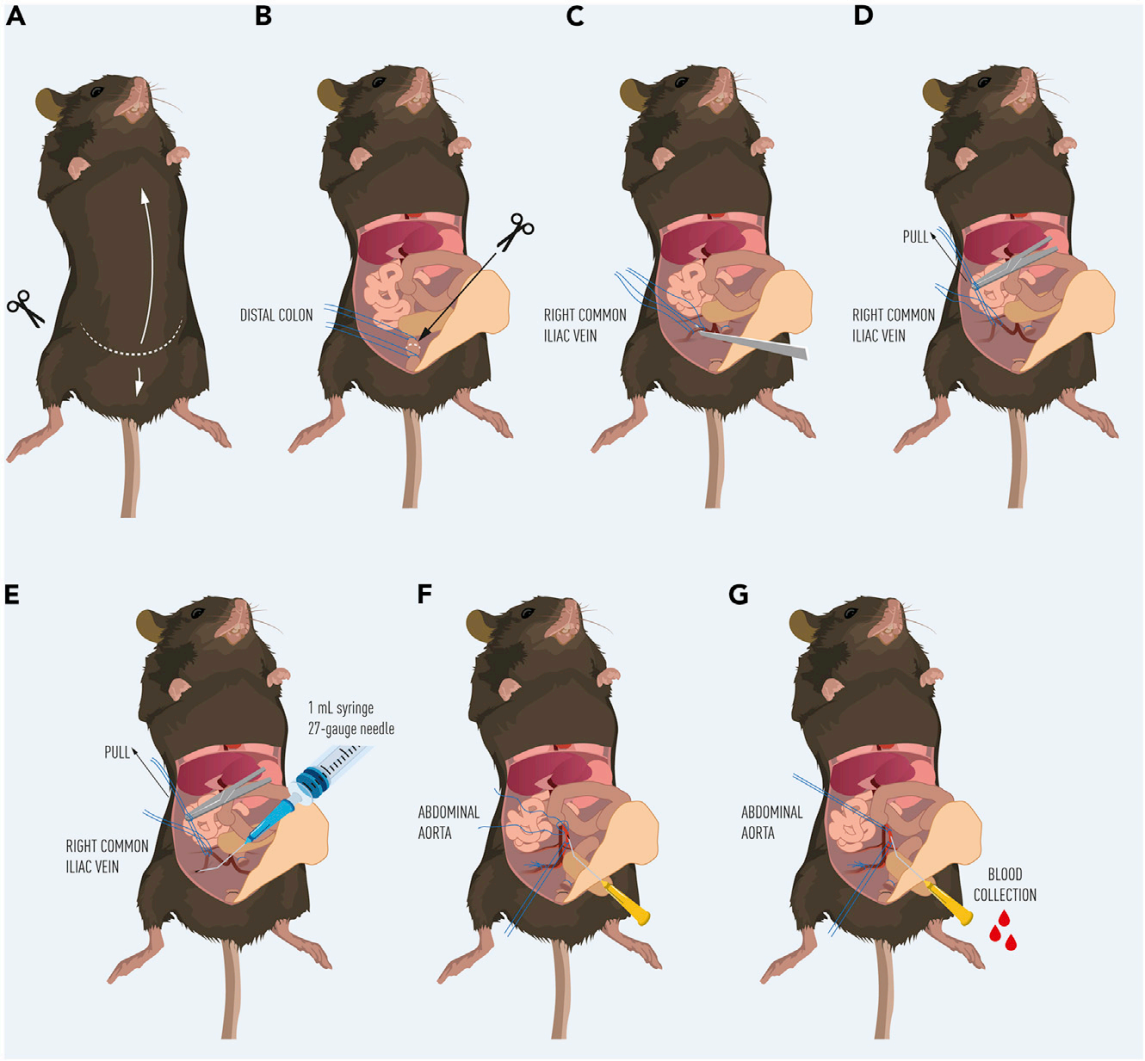
Hindlimb blood sampling from the right common iliac vein (A–E) and abdominal aorta (F–G)
31. Locate the distal colon by moving the intestines and gonadal adipose tissue to the side.
32. Tie two ligatures around the colon (Figure 2B).
33. Cut the colon in-between the two ligatures (Figure 2B).
34. Locate and expose the right common iliac vein by gently removing connective tissue from the area using forceps and cotton swabs (Figure 2C).
35. Put two ligatures around the right common iliac vein and leave the ligatures loose (Figure 2C).

***Note:*** One ligature for pulling back to create tension and one to quickly tie off to avoid blood loss after the blood sample has been taken (Figure 2C).

25. Load a 1 mL syringe with pentobarbital saline solution (50 mg/mL).
26. Scruff and inject the mouse intraperitoneally with pentobarbital solution (100 mg/kg).
27. Return the mouse to an empty clean cage. Anesthesia should take approximately 3–5 min.
28. Once under anesthesia, place the mouse supine on the dissection station.
29. Using forceps, pinch the paw to check for a reflex response. If there is no reflex response, proceed to next step. If a response is obtained, wait until there is no response.
30. To open the mouse, make an incision with scissors at the base of the abdomen. Cut upwards up to the ribcage (Figure 2A).

36. Using clamped forceps, gently pull the proximal ligature to isolate the right common iliac vein and provide tension on the vein (Figure 2D).
37. Using a 1 mL syringe and 27-gauge needle, collect a 60–80 μL blood sample from the right common iliac vein (Figure 2E).
38. Remove the needle from the syringe and decant the blood sample into a Microvette® blood collection tube containing EDTA. Place the tube on ice.
39. Tighten the ligatures around the right common iliac vein to avoid significant blood loss as soon as possible after blood sampling.
40. Locate and isolate the abdominal aorta using forceps and cotton swabs (Figure 2F).
41. Loosely tie a ligature around the abdominal aorta (Figure 2F) and another loose ligature around the aorta as distally as possible to provide tension.
42. Insert catheter into the abdominal aorta (Figure 2G) and then tighten the ligature to prevent the catheter from falling out.
43. Sample 160–180 μL of blood via the catheter into a Microvette® blood collection tube containing EDTA. Place the tube on ice.

***Note:*** The blood from the catheter should run freely into the tube without the need for attaching a syringe.

44. Dissect the *gastrocnemius* muscle, rinse in phosphate buffered saline and flash freeze for later analyses.

***Note:*** A more detailed approach to the proper dissection of the *gastrocnemius* can be found here.^4^

45. Spin the obtained blood samples at 2,000 g at 4°C for 10 min.
46. Remove the plasma supernatant and decant into a 0.2 mL tube. Store at −80°C for further analysis.

## Expected outcomes

The successful application of the above protocol will result in obtaining plasma samples that can be utilized to quantify the net uptake and release of metabolites and other targets from both the liver and hindlimb muscles. Regarding the liver sampling, most of the blood supplied to the liver comes from the portal vein and is representative of the blood that drains from the abdominal organs. Therefore, the portal vein is the most appropriate site for sampling blood coming to the liver since it represents a mixed contribution from the various organs. In turn, the liver drains via the two hepatic veins into the upper part of the caudal vena cava. The sampling catheter is placed in the cranial vena cava via the heart and since it is obstructing, there is no chance that blood will be drawn retrogradely from the cranial vena cava or from the heart. Furthermore, the ligature around the caudal vena cava (above the level of the kidneys) ensures that blood from the upper part of the caudal vena cava, where we take the samples, exclusively drains from the liver. Finally, a proper dissection will yield liver and *gastrocnemius* muscle samples that can be utilized for further analyses.

For blood sampling across the liver, an average expected yield of plasma is 80 μL from the portal vein and 40 μL from the vena cava. For sampling across the hindlimb, an average expected yield of plasma is 60 μL from the right common iliac vein and 90 μL from the aorta.

## Limitations

### Treadmill associated stress

The use of forced treadmill running results in a significant amount of stress for the animal. The stress is caused by the use of negative stimuli such an electric shock grid or intervention from the researcher via handling. Therefore, when designing treadmill-based experiments, it is important to ensure that sedentary mice are placed upon a stationary treadmill for the same duration as running mice and handled by the investigator. Doing so will mitigate some of the influence of stress in downstream analyses.

Furthermore, for ethical reasons, some prefer alternative strategies to the electric shock such as blowing air on the mouse or gently brushing the back and tail of the mouse during the running bout.^7^ Differences in the strategy to motivate the mice to run may introduce variation. Therefore, it is important to consider how the mice will be motivated to run before the experiment takes place. Furthermore, if the protocol requires that mice are to be run until exhaustion, a stringent endpoint needs to be employed such as time spent on the shocker.

## Troubleshooting

### Problem 1

Poor running performance during acclimation to the treadmill or treadmill exercise bout. Some mice will exhibit poor running performance either by spending too much time on the shock grid or failing to run in the correct direction.

### Potential solution

For some mice, it may be necessary to perform additional acclimation bouts. If running performance is particularly poor, it be necessary to exclude the mouse from the experiment. However, these occurrences are rare and can be mitigated by ensuring a suitable sample size before the experiment begins.

### Problem 2

Ruptured blood vessel during hepatic or hindlimb blood sampling. Given the size of the blood vessels to be sampled from, it is likely that some of the vessels will rupture during sampling.

### Potential solution

Bleeding from a ruptured blood vessel may be stopped by applying sterile gauze. If bleeding cannot be stopped, then the animal should be terminated. Given the technical challenges of the above protocol, it is important to plan for such events when deciding upon the sample size.

Sufficient prior practice should mitigate these events.

## Resource availability

### Lead contact

Further information and requests for resources and reagents should be directed to and will be fulfilled by the lead contact, Juleen R. Zierath (juleen.zierath@ki.se).

### Materials availability

This protocol did not generate any unique reagents.

## Data Availability

Original data have been deposited into Mendeley Data: https://doi.org/10.17632/6x5vd4d5rd.1.
